# Waiting reasons affecting the handling process at liquid bulk terminals

**DOI:** 10.1186/s41072-022-00109-6

**Published:** 2022-04-22

**Authors:** Eren Salihoglu, Elif Bal Beşikçi

**Affiliations:** 1 Maritime Transportation Engineering Ph.D. Programme, Institute of Science and Technology, Istanbul Technical University, Istanbul, Turkey; 2 Department of Maritime Transportation Management Engineering, Istanbul Technical University, Istanbul, Turkey

**Keywords:** Liquid bulk cargo, Handling, Terminal, Port, Efficiency, Reasons for the waits

## Abstract

Since liquid bulk cargoes are the most frequently handled cargo types in Turkish ports in 2019 and the latter in 2020, the loading and unloading times of liquid bulk cargoes are important in terms of handling efficiency. While a higher amount of cargo handled per unit time increases the profitability for terminal operations, the short handling time of tankers at the terminal provides an advantage for the next voyage. In this study, the process in the terminals in Turkey where 19.05% of the total liquid bulk cargo handled was reviewed as well as the reasons for the waits in this process. Thus, it was aimed to find subjects that need to be focused on to reduce the waiting times. An expert team was asked to indicate the reasons for the waits using the fishbone method. In addition, a questionnaire was applied to a sample group of 134 people who were the stakeholders of the handling process. It was found out that the reasons for the waits obtained through the questionnaire matched those determined by the fishbone method. To reach a solution, prioritization was provided by scoring the reasons that lead to waits.

## Introduction

Crude oil is one of the non-renewable energy sources. It is extracted as raw material only from certain regions across the world. Therefore, it is transported by sea from the oil-producing countries to the supplier countries to be processed. In addition, the finished products manufactured in the refineries of countries that are not producers of raw materials are mostly transported to world markets by sea. 2.605 billion tons of international maritime trade in 1970 reached up to 11,076 billion tons in 2019. Along with general cargo ships and container ships, tankers are used in maritime transportation and carried 3.169 billion tons of liquid bulk cargo in 2019 which is equal to 28.6% of the world’s maritime transportation (UNCTAD 2020).

The COVID-19 pandemic, which started at the end of 2019 and took a heavy toll on countries all across the world, has had severe and negative impacts on international trade flows. The curfews and restrictions on travel imposed as per the measures taken on a global scale, the widespread use of remote work and telework, and people staying home except for essential activities due to the pandemic resulted in a decline, especially in global oil demand. In 2020, as the devastating effects of the pandemic began to be felt, the volume of trade fell compared to 2019 in many sectors such as agriculture, automotive, textile, and energy. According to April 2020 data of the United Nations Conference on Trade and Development (UNCTAD) (UNCTAD 2021), the most significant decline was experienced in the automotive sector (− 49%), followed by the energy sector (− 39%). All these developments had a negative impact on maritime trade. Ships carrying liquid bulk cargo worldwide increased by 2.5% in the first quarter of 2020 compared to the same period in 2019, while the number fell by 6.3% in the second quarter when drastic COVID-19 measures, such as shutdowns, border closures, and flight restrictions, were implemented (UNCTAD 2021).

According to the handling statistics at Turkish ports, the liquid bulk cargo handled in 2019 was 155,253,914 tons, 32.06% of the total handled (Ministry of Transport and Infrastructure 2019). This figure also expresses the most handled cargo type. In 2020, the decline in demand for liquid bulk cargo due to the COVID-19 pandemic also had a negative impact on the handling quantities at the ports, and the total liquid bulk cargo handled fell by 5.54% to 121,710,948 tons. In 2020, the liquid bulk's share of total handled cargo fell by 2.53–29.52% (Ministry of Transport and Infrastructure 2020).

The decline in the amount of handling both on a global scale and in Turkey affected the profitability of the ports. Therefore, projects aimed at improving handling efficiency gained more importance. In this study, the processes in two terminals in Turkey were analyzed where 26.05% of total liquid bulk cargo handled in 2019 and 19.05% of total liquid bulk cargo handled in 2020. To identify the reasons for the waits that affect the efficiency of the handling process of the tankers negatively, data were collected from the expert team composed of terminal managers using brainstorming technique and fishbone method. In addition, the reasons for waits were scored through a questionnaire conducted to terminal employees, ship personnel, and inspector company employees.

## Literature review

It was found that studies on port efficiency in the literature were generally conducted on efficiency and productivity analyzes of ports. However, data envelopment analysis was also used frequently and studies were conducted specifically on container ports.

Çağlar and Oral studied the concepts of productivity and efficiency and aimed to make a comparative analysis of port productivity and efficiency measurement methods in national and international studies. To that end, they reviewed the studies in the literature conducted in the last 20 years (Çağlar and Oral 2011).

In their study, Clark, Dollar, and Micco studied the determinants of the cost of shipping to the United States using a database on the shipment of products from various terminals and ports around the world. They found that increasing the productivity of a port from 25 to 75%, decreased the shipping cost by 12% (Clark et al. 2001). The authors listed lots of countries’ port efficiency indexes in their study. They measured Turkey’s port efficiency score between 1 and 7, and determined it as 3.81.

Wanke, Nwaogbe, and Chen analyzed the handling data of six major Nigerian ports from 2007 to 2013 for assessing the efficiency by applying a two-stage fuzzy-based methodology. At the first stage, fuzzy data envelopment analysis models for conjectures with respect to scale returns were used to determine the efficiency of Nigerian ports. In the second phase, fuzzy regressions were used to determine the relationship of a set of contextual variables related to port service level, berth utilization, accessibility, cargo type, and operator type on port efficiency. The results showed that operator and cargo type had an impact on efficiency levels (Wanke et al. 2018).

In his study, Akyürek aimed to analyze the efficiency of important ports in the Black Sea Region compared to Karadeniz Ereğli Port between the years 2010–2013 and used data envelopment analysis as the method. In the study, the population density of the cities, the coastal area, and the number of ports in the Black Sea Region constituted the input of the analysis, while the output was formed by the gross tonnage of all ships visiting city ports and the amount of cargo handled at the ports (Akyürek 2017). Sağlam et al. (2018) suggested minimizing the duration of each ship's stay in port to increase the efficiency of the ports. To that end, they analyzed a port that received port efficiency investment using berthing time difference (BTD) as output through data envelopment analysis (DEA).

Park and De (2015) focused on reviewing approaches to performance measurement and provided an examination of the applicability of alternative (four-stage) Data Envelopment Analysis to seaport efficiency measurement. The authors found that the alternative DEA was a potentially powerful approach to assessing the overall efficiency of ports.

According to Güner's study, a two-stage model was developed by data envelopment analysis, which is the most widely used method to measure port efficiency. In the first stage of the model, it was aimed to maximize the cargo handled with the available resources and the number of served ships, while in the second stage, the aim was to generate maximum revenue from the handled cargo served vessels (Güner 2015).

On the other hand, Temiz et al. (2018) made the efficiency analysis of Samsun Port and studied importance of various factors as well as their relationship with eachother affecting port operational performance through a fuzzy Decision Making Trial and Evaluation Laboratory (DEMATEL) method (Temiz et al. 2018).

In their study, Eliiyi et al. (2008) discussed the berth allocation problem, which is an important stage for the efficiency of ports. In addition, the impact of the berth allocation on other processes was emphasized, studies in the literature were reviewed, and suggestions were offered regarding the model that can be applied in Turkey (Eliiyi et al. 2008).

Cullinane and Wang used the DEA approach for measuring the efficiency of container terminals in Europe in their study. Relative efficiency estimates were derived for a sample of 69 container terminals in Europe that handle over 10,000 twenty-foot equivalent units (TEU) annually. The container terminal production’s scale characteristics were also considered part of the study, as is the relationship of efficiency with regards to geographical influence (Cullinane and Song 2006).

Ateş and Esmer aimed to analyze the relative efficiency of the container terminals in Turkey based on 2012 data using data envelopment analysis and Free Disposable Hull technique. In addition, they determined the efficiency ranking of container terminals in Turkey by applying super-efficiency models (Ateş and Esmer 2014). In another efficiency analysis of container terminals, Sarıoglu and Ozdemir measured the efficiency of container operations using the simulation method. To that end, two models were developed. While the current situation was reflected in the first model, container loading and unloading operations performed by quay crane were simulated independently of the transport vehicle in the second model. Thus the objective was to observe the waiting time of the cranes for the transport vehicle (Sarıoğlu and Özdemir 2018). In his study, Görçün proposed Black Sea ports to be used for making efficiency and productivity analysis using a model created by the integration of entropy and EATWOS methods (Görçün 2019).

Cullinane and Wang applied the data of the world's largest container ports using Data Envelopment Analysis (DEA) and Stochastic Frontier Analysis (SFA) and compared the results obtained (Cullinane and Wang 2006).

Esmer et al. (2010) analyzed the cargo handling in container ports in Turkey and aimed at simplifying container handling equipment for greener ports. In this study, the number of optimum cargo handling equipment damaging the environment least was obtained through simulation.

Tongzon (1995) focused on quantifying the relative contributions of various factors influencing a port's performance and efficiency. The author established a model for port performance and efficiency for determining them with empirical tests with various factors. The study contained 23 international ports as a sample. By the way, the study was able to provide an empirical basis for the critical role of port efficiency relative to other factors in the overall port performance.

Esmer et al. (2013) examined the İzmir Alsancak port quay and modeled the arrival and berthing conditions of the ships through the Arena simulation program. Thus, they determined performance criteria for ships such as the waiting times before berthing, the average mooring time, and the average number of ships in the queue. In their studies, Kaffka et al. (2012) stated that the processes in container terminals had interconnected and complex structures. The aim of the study was to develop a simulation model to determine the relative effects of the processes due to their stochastic nature. Using a simulation package called ContSim, they aimed at optimizing the terminal by identifying the best mix of operating strategies for crane control, stacking area, handling area, and resource management for every system load that can be handled by the terminal. In another study, Zeng and Yang (2008) developed a simulation optimization method for scheduling loading operations in container terminals.

## Research methodology and findings

The handling process in the terminals that will be covered in this study includes many procedures. Therefore, the handling process is divided into parts which contain the process from issuing the notice of readiness document by the tankers for berthing at the inspected terminals to completing the loading or unloading operations and departing. A team of specialists consisting of managers working in the operation, logistics, and planning departments of the relevant terminals was formed. This team had a brainstorm and identified the factors reducing the handling efficiency using the fishbone diagram. In addition, the factors identified in the fishbone diagram were scored through an online questionnaire conducted to the terminal employees, ship personnel, and inspectors involved in the handling process. Thus, the factors affecting the handling efficiency were prioritized and focus areas were identified to find solutions in terminals. Basic data such as name, location, handling volume, and equipment of the terminals were kept secret in terms of preservation of trade secrets.

### Definition of subprocesses

The defined operations throughout the processes to be reviewed are grouped as anchoring, berthing at the pier, preparation to handling, loading/unloading, post-handling procedures, and departing from the pier. Terminals will be referred to as terminals A and B.

- Anchoring: It refers to the period a tanker lies at anchor and waits for berth after issuing the notice of readiness document when there is no available loading or discharging berth.
- Berthing at the pier: It includes calling the tanker to the allotted space at the pier, embarkation of the pilot to the ship, and berthing maneuvers of the tanker with tugs.
- Preparation to handling: It includes the preparations until the beginning of loading/unloading, such as sampling upon completion of berthing, signing of various statutory controls and protocols (security declaration and customs controls, signing of the ship/shore safety checklist), and connecting the load flow arm.
- Loading/unloading: It consists of loading and unloading operations performed for handling the cargo, including sampling, line displacement method that is used to record and compare volumes delivered to volumes received, and operations performed during the switch between different cargoes.
- Post-handling operations: This process includes closing the relevant valves and dismantling the loading arm upon the completion of the loading/unloading operation.
- Departing from the pier: It includes departing from the pier once the pilot comes aboard and tugboats move the ship upon the completion of various documentation procedures.

The waits experienced during the subprocesses mentioned above having a negative impact on the efficiency in the handling process were identified using the brainstorming method and categorized by the fishbone method.

### Brainstorming and fishbone methods

The brainstorming technique was used for the first time by an advertiser named Osborn in the creation of new brand names and slogans for new products in 1957 (Osborn 1957). Brainstorming is a group discussion method intended to develop problem-solving skills and develop creative ideas of individuals collaboratively (Nakiboğlu 2003). In this technique, generating more creative ideas in numbers is considered important regardless of whether the ideas make sense. Groups try to generate a large number of creative ideas on the relevant subject in a short period of time through brainstorming technique. A large list of ideas is created by the ideas generated and then opened to discussion, which may eventually be funnelled down to a smaller list of priority items.

**Fig. 1:**
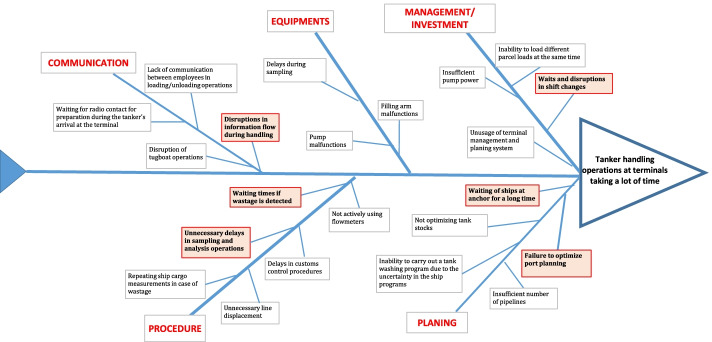
Application of fishbone diagram

The Fishbone diagram, also known as the cause and effect diagram, was first applied in 1953 by Kaoru Ishikawa (Akgemci and Güleş 2010). The fishbone diagram is a technique that helps to identify possible causes of a problem. In addition, it provides support to identify and improve the factor that has the most impact on the result (Ishikawa 1991; Çubukçu 2020). The literature review of the studies on process development showed that there are several studies in which the problems of the businesses are identified through brainstorming technique and then possible causes of those problems are identified through fishbone diagram (Deste and Berber 2018). In this study, the problems leading to waits were identified through the brainstorming technique and the causes of these problems were identified using the fishbone diagram. The resulting diagram of the application is given in Fig. 1.

Accordingly, the definition of the problem was first made to the participants as “longtime handling operations at the terminals”. Then, the factors leading to the problem were listed through the brainstorming technique. The 23 factors identified were grouped under 5 main headings as management/investment, equipment, communication/personnel, procedure, and planning. The scores of the participants showed that the following 6 factors had a significant influence on extending the duration of the tanker's handling process more than the others.

- Waits during the shift change of the employees working in maritime operation unit of the terminals,
- Disruptions of the flow of information experienced due to the lack of communication or the inability to communicate effectively during the handling process,
- Waits in case of any wastage occurrence in consequence of calculating the cargo upon the completion of loading/unloading,
- Unnecessary delays in sampling and sample analysis,
- The time ships spend waiting on anchor for berths due to the density at the piers
- Lackness and failures in optimization of port planning which is about berthing time of ships.

### Questionnaire

The questionnaire was conducted to a sample of terminal employees working in maritime operation departments, shipmasters who are tanker crews, and inspector company employees operating on tankers. Tanker crew consisted of maritime company employees hired by terminals on a timely basis. Therefore, they had knowledge and experience about waiting times due to the frequent visits made to the terminals. The sample consisted of a total of 134 people, including employees working in the maritime operation unit of the terminals, shipmasters, and inspector company employees. Formula 1 was used to identify the sample (Cloudresearch 2021). Accordingly, the population determined with a 5% margin of error for a 95% confidence interval consisted of 100 individuals.

1 $$Sample size = \frac{{\frac{{z^{2} \times p\left( {1 - p} \right)}}{{e^{2} }}^{ } }}{{1 + \frac{{(z^{2} \times p\left( {1 - p} \right)}}{{e^{2} N}}}}$$

N = Sample Size, e = Margin of Error (percent in decimal format), z = Z value, p = Percentage picking a choice.

The employees involved in the sample were in different locations and face-to-face meetings were not possible due to the COVID-19 measures. Therefore, an online questionnaire was decided to be conducted due to the accessibility to many people in a short time and the method's ease of application. The problem was defined in the introduction of the questionnaire and the participants were asked to score the reasons of wait in the handling process of the tankers. The questionnaire consisted of 13 questions. The first 4 questions were generated to understand the demographic characteristics of the participants while the next 7 questions were generated to determine the waits that negatively affect the handling process and handling performance of the terminals. They were generated using a Likert scale. The last two questions were open-ended, which aimed at learning what needs to be done to avoid the waits.

A preliminary test was conducted to the expert team whose opinions were sought using the fishbone method and brainstorming technique to give the final version to the questionnaire. The preliminary test was performed as an online interview. Feedback was received from the participants regarding the clarity and scope of the questions in the preliminary test. As a result of the preliminary test, the Cronbach's alpha of the measurements was found to be 0.902. The feedback received enabled to make editing changes in some expressions of questions.

The Cronbach's alpha coefficient calculated upon the completion of the research was found to be 0.936. According to the calculated value (0.936), the reliability was found to be high (0.80 ≤ α < 1.00) (Kalaycı 2008).

It was learned that 41% of the participants had more than 10 years of experience, and 37% of them had 5–10 years of experience regarding the question about the participants' professional experience, which was selected among all questions to measure the demographic characteristics of the questionnaire. In this respect, the participants were observed to have sufficient experience in the field they worked.

In the next 5 questions, the participants were asked to score the factors that negatively affect the handling process. While these questions were generated, the handling process was divided into parts, the reasons for the waits in each part were scored and the extent of effect was tried to be learned.

A Likert scale was generally used to survey awareness, feelings, experience, and behaviours. It consisted of a series of statements that respondents may choose from to rate their responses to evaluative questions (Vagias 2006). A 5-point Likert scale was used as the score scale. The scale definitions based on the scores are given in Table 1.

**Table 1:** Definition of the scale used in the questionnaire

Score	Definition
1	No affect
2	Minor affect
3	Neutral
4	Moderate affect
5	Major affect

**Fig. 2:**
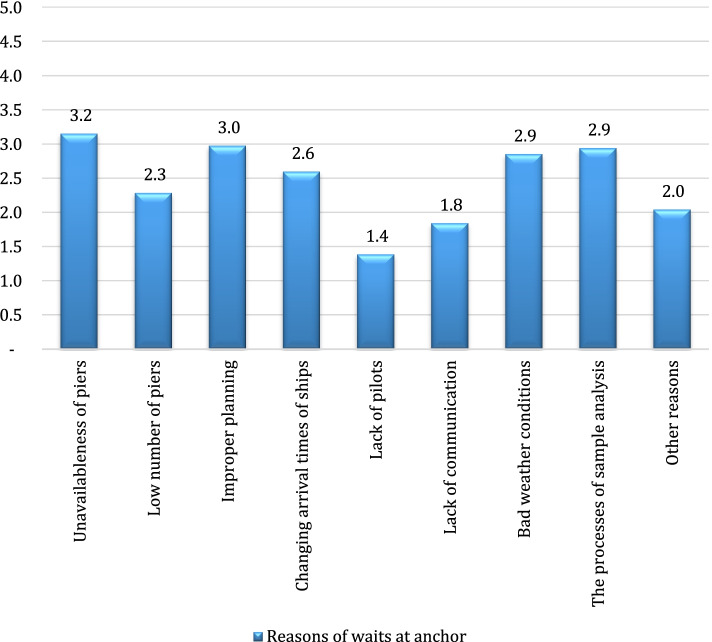
Reasons of waits at anchor

The outcomes of the question about the reasons of the waits experienced by the ships which were directed to anchor prior to berthing at the terminals are shown in Fig. 2.

**Fig. 3:**
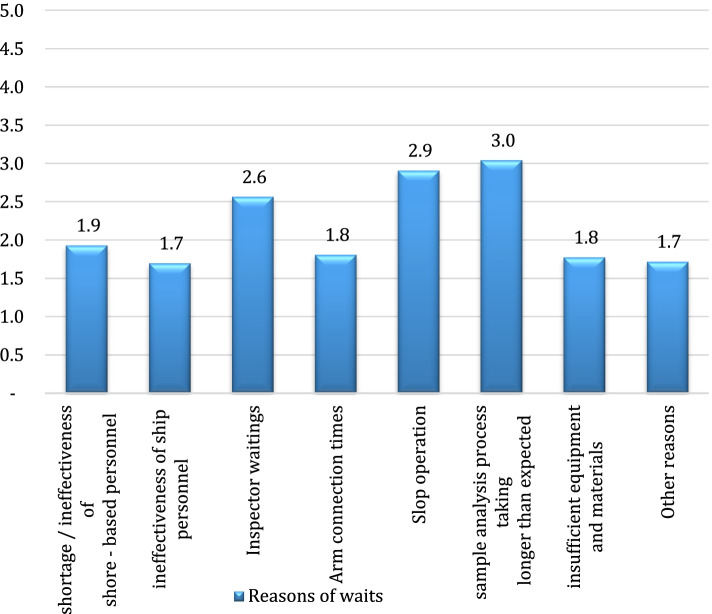
The reasons for the waits during the preparation period before handling

Accordingly, the most significant reason for the waits at anchor was found to be the unavailableness of piers in the terminals and improper pier planning. The outcomes of the sixth question are shown in Fig. 3, in which the reasons for the waits during the preparation to loading/unloading of tankers were scored,.

**Fig. 4:**
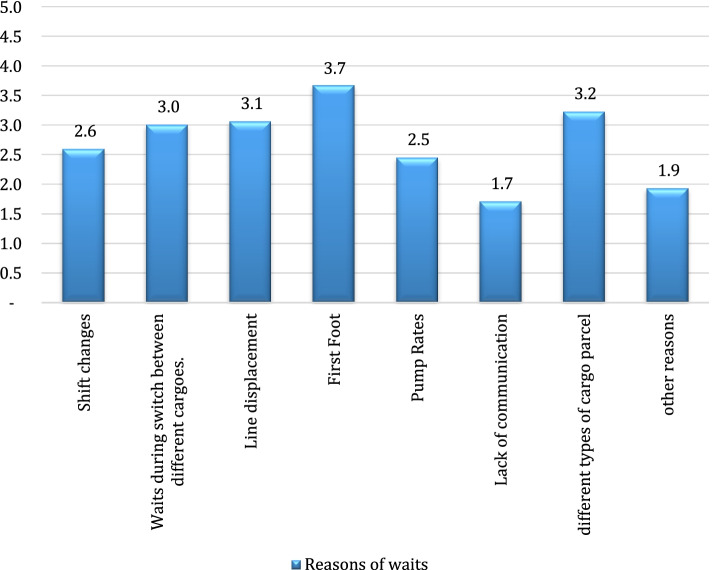
Reasons for the waits during loading/unloading operations

Accordingly, the most significant reasons for the waits during the preparation to handling were found to be “the sample analysis process taking longer than expected”, “slop operation” and “inspector related waits”. The scores given to the reasons of waits experienced during loading or unloading operation are shown in Fig. 4.

**Fig. 5:**
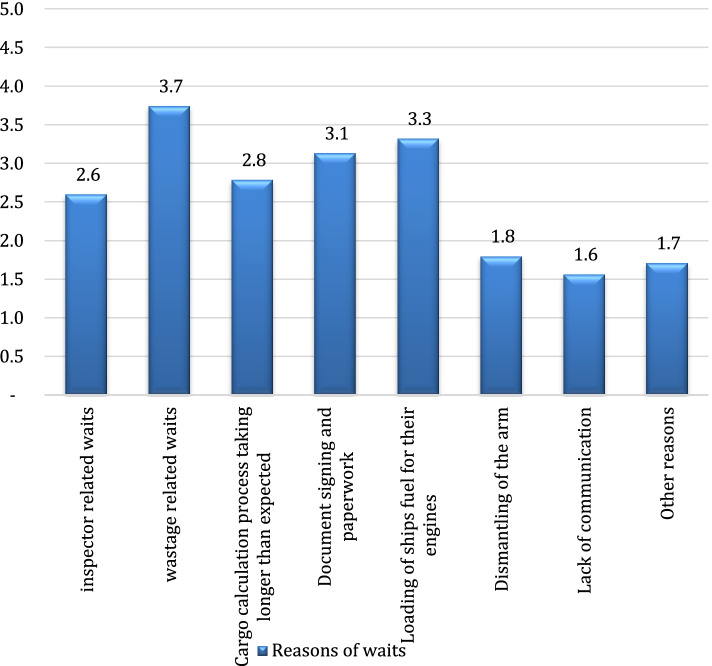
Reasons for the waits upon the completion of loading or unloading operations

The most significant reasons for the waits during loading or unloading operations were found to be “the first foot operations taking longer than expected”, “loading cargo in different parcels in the same tanker” and “applying line displacement process”. The reasons for the waits experienced upon the completion of loading or unloading operations of tankers are shown in Fig. 5.

The most significant impact on the waits experienced upon the completion of handling operations was found to be the process taking longer than expected due to the wastage in consequence of calculating the cargo. In addition, deferring the supplying of fuel (bunker fuel) to be used by the propulsion system of tankers until after handling operation as well as the paperwork taking longer than expected after the handling process had a significant role in reducing the handling efficiency.

**Fig. 6:**
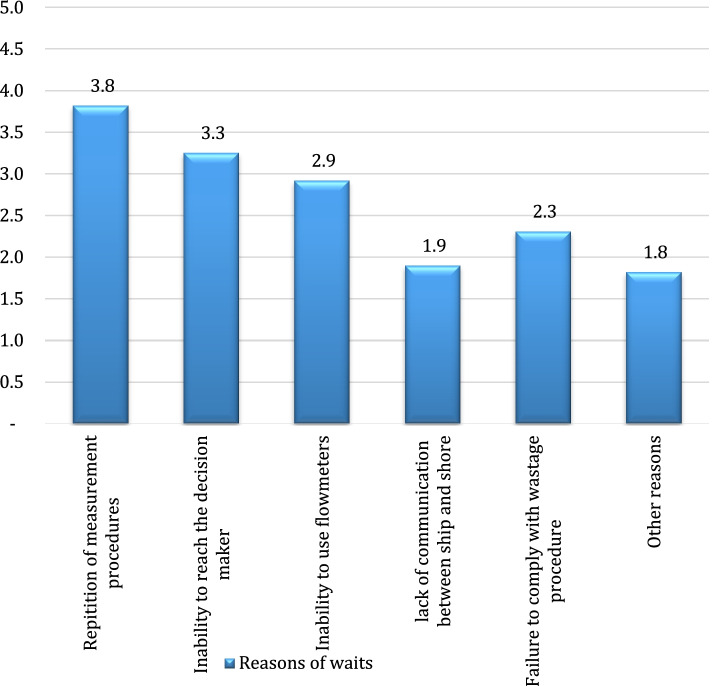
Reasons for the waits in case of wastage occurrence

The answers given to the question in which “reasons for the waits at the time of wastage” were asked in the scale of reasons obtained from the brainstorming and fishbone diagram were shown in Fig. 6.

The most significant impact on the reasons of the waits in case of wastage occurrence was found to be the repetition of measurement procedures. In addition, the inability to reach the decision-maker and to use the flowmeters had a significant role in the occurrence of the waits. The handling performances of terminals A and B were found to be 3.96 and 3.45 respectively in questions asked to evaluate the handling performance of terminals on a scale (from 1 (very good) to 5 (very bad)). Open-ended questions were directed to the participants about which problem needed to be solved to improve the handling efficiency at the terminals, and the answers received were grouped. These answers generally show similarity to the choices found and scored in the previous questions. In this context, the issues required to be resolved in terms of Terminal A are as follows. Not making a proactive pier planning, long document waiting times, waiting times in sample analysis processes, the time lost due to the shift changes of terminal employees, the time lost to reach an agreement in case of wastage occurrence, and key performance indicators (KPI) specific to processes. The issues required to be resolved for terminal B are grouped as follows. Occurrence of wastage usually during loading operations on the same tankers, frequent failure of the circulation pumps at the piers, keeping low pump transfer rates for some products, the time lost due to repetition of measurement process more than twice in case of wastage occurrence, and the inability to reach the authorized decision-makers.

## Discussion

In this study, the process in two terminals in Turkey was reviewed where a significant amount of liquid bulk cargo was handled. The expert team composed of terminal managers identified many factors reducing handling efficiency. The team created a fishbone diagram by prioritizing the most influential factors. The literature review showed that several studies used data envelopment analysis in efficiency measurement (Güner 2015; Akyürek 2017; Ateş and Esmer 2014; Çağlar and Oral 2011; Park and De 2015; Cullinane and Wang 2006). However, in this study, expert opinion and questionnaire methods were used to identify the waits affecting the efficiency of terminals examined. Thus, a solution-oriented approach was tried to be shown through identifying and prioritizing the unnecessary waits.

In the studies conducted to the expert team, it was stated that not too much wastages were encountered during loading/unloading operations. However, it was highlighted by the same team that the process of problem-solving took too long in case of wastage occurrence. The reasons for wastage occurrence were scored, and it was found that "the Repetition of measurement procedures" had the highest score. The interview with the expert team revealed a new practice in which the measurements previously taken were repeated in case of wastage occurrence. If the result remained the same, the measurement was taken for the third time. It was considered that repetition of the measurement process did not serve the purpose, caused a loss of time, and thus reduced the handling efficiency. This revealed the necessity of conducting root cause analysis regarding wastage issue and standardizing the process procedurally.

In terminals where liquid bulk cargo is handled, unlike handling of containers and dry bulk cargo, there are procedures such as sampling, sample analysis, and line displacement measurement application. The studies with the expert team showed that unnecessary waits occurred during the first-foot sample collection and delivery of the sample to the relevant laboratory. Loading/unloading operations did not continue unless the analysis results were reported to the terminal. Therefore, waiting times had a negative impact on the whole process. Questionnaire results also showed that the most significant factor causing inefficiencies in the loading/unloading process was the "first-foot analysis processes".

The outcomes of the questionnaire showed that the most significant reason for directing ships to anchor was the unavailableness of piers in the terminals and improper pier planning, which revealed the requirement for conducting an optimization study on piers. A future study will enable to reduce the times at anchor and handle more cargo through optimization.

This study was conducted on the process in two terminals in Turkey where significant amount of liquid bulk cargo were handled. Considering that each terminal designs its own handling process and manages the total process in accordance with its own procedures, each terminal needs to conduct similar studies on terminal handling efficiency based on its dynamics.

Although this is not examined in this study, the Vessel Traffic Services (VTS), where the crowded traffic of ships are managed, and the crews who manage and direct maneuvering of the ships to the terminals have a great effect on the anchoring or mooring of the ships (Baldauf et al. 2019). In this respect, the effects of the mentioned factors on efficiency can be examined.

Over the years, the further development of technology has clearly resulted in bigger ship structures, adding more machinery and navigation systems, better loading types of equipment, and sensors (Dalaklis et al. 2020). The fact that all these technological developments work in harmony in terms of both the ship and the port will also affect the handling efficiency. Today, when autonomous ships and terminals are spoken, the importance of trained human resources and their training becomes even more evident (Baldauf et al. 2018). Various processes are managed by remote monitoring and control, both on the ship and at the terminals. Well-trained operators using all these systems will directly affect handling efficiency. In the future, another study can be conducted on the advancement of technology,the development of human resources, and the handling efficiency of terminals.

## Conclusion

COVID-19 pandemic took a heavy toll on countries all across the world and had severe and negative impacts on economies. The maritime industry also took its share of this impact. Despite a positive effect in some areas, a decline of 7.4% was observed in 2020 compared to 2019, especially in the liquid bulk cargo category (TÜRKLİM 2021). This situation also had an impact on the profitability of the ports and once again revealed the significance of the handling efficiency.

In this study, the process in two terminals in Turkey was reviewed where a significant amount of liquid bulk cargo was handled. The expert team consisting of terminal managers identified 23 factors that had a significant role in reducing the handling efficiency and selected 6 of them as the factors causing this problem. These factors identified are “Delays due to the shift change of terminal employees”, “disruptions of the flow of information”, “lack of communication or the inability to communicate effectively throughout the process”, “sample analysis process taking longer than expected, “waits in case of any wastage occurrence, and “unavailableness of piers in the terminals due to the density and improper pier planning”.

In a questionnaire conducted to a sample group including seafarers, inspectors, and terminal employees, the factors that had a significant role in reducing the efficiency were scored and "the unavailableness of piers", "sample analysis process taking longer than expected", "unnecessary waiting times during the first foot process" and "waits due to wastage" had the highest score. Accordingly, the data obtained from the brainstorming technique and fishbone method based on expert opinion matched those obtained from the questionnaire. Thus, the topics that need to be studied to improve the handling efficiency of the terminals were determined. Conducting similar studies in ports where different types of cargo are handled will contribute to the development of the Turkish port sector.

## Data Availability

The datasets analysed during the current study are not publicly available due to termianals and companies commercial confidentiality but are available from the corresponding author on reasonable request.

## Abbreviations

BTD: Berthing time difference
ContSim: Container terminal management with simulation
DEA: Data envelopment analysis
DEMATEL: The decision making trial and evaluation laboratory
EATWOS: Efficiency analysis technique with output satisficing
KPI: Key performance indicators
TEU: Twenty foot equivalent unit
UNCTAD: United Nations Conference on Trade and Development
VTS: Vessel traffic service
